# A Study of Clinical Profile, Radiological and Electroencephalographic Characteristics of Sporadic Creutzfeldt-Jakob Disease From a Tertiary Care Hospital

**DOI:** 10.7759/cureus.50008

**Published:** 2023-12-05

**Authors:** Komal Usha C Madineni, Naveen Prasad S V, Vengamma Bhuma

**Affiliations:** 1 Neurology, Sri Venkateswara Institute of Medical Sciences, Tirupati, IND

**Keywords:** periodic sharp wave complexes, clinical characteristics, electroencephalography, magnetic resonance imaging, creutzfeldt-jakob disease

## Abstract

Background

Sporadic Creutzfeldt-Jakob disease (CJD), the most common form of human prion disease, is the archetypal diagnosis in this category. However, the spectrum of possible diagnoses is wide, encompassing various treatable conditions. A lack of standardized diagnostic criteria and a tendency to opt for brain biopsies and clinical autopsies can be limiting factors in reaching a conclusive diagnosis.

Objective

This study aims to retrospectively analyze clinical and investigative findings in patients referred to a specialized neurology clinic exhibiting rapidly progressive dementia. These patients were ultimately diagnosed with Probable sporadic Creutzfeldt-Jakob disease (CJD) based on the 2018 CDC criteria for sporadic CJD.

Materials and Methods

This study included cases of CJD diagnosed based on clinical, electrophysiological, and imaging parameters at a tertiary care hospital in India from 2016 to 2020. The diagnostic criteria proposed by the CDC (Centers for Disease Control and Prevention) were employed to categorize patients as definite, probable, or possible CJD cases. All patients underwent MRI (magnetic resonance imaging) imaging and EEG ( electroencephalography) recording, while diagnostic brain biopsies were not conducted due to a lack of consent from close relatives.

Results

This observational descriptive study comprised four patients diagnosed with Probable sporadic CJD (sCJD), all of whom were female. The patients exhibited an age range of 57 to 75 years at the onset of the disease, with a mean age of onset at 67.5 years. Unfortunately, all patients succumbed to the disease within 6 months of its onset. Rapidly progressive dementia was a common symptom in all cases. Additionally, patient one and patient four displayed myoclonus and dystonia, patient two exhibited myoclonus and akinetic mutism, and patient three had myoclonus, chorea, and ataxia. MR brain imaging, including T2 sequence, FLAIR sequence, and DWI/ADC mapping, was performed on all patients, revealing both cortical gray matter and deep gray matter (basal ganglia) T2/FLAIR hyperintensities with DWI restriction. A cortical ribboning pattern was observed in all cases. EEG results indicated generalized delta slow waves with triphasic complexes in three patients, while patient three alone displayed periodic sharp wave complexes at a frequency of 1 per 1 - 1.5 seconds.

Conclusion

MRI with DWI and ADC brain mapping emerges as the most valuable diagnostic tool for patients with clinical presentations suggesting sCJD. In this study, all patients displayed restricted diffusion, as confirmed by ADC mapping. Regrettably, the characteristic features of sCJD with restricted diffusion in the cortex, thalamus, and basal ganglia may often elude detection by radiologists outside specialized centers, resulting in diagnostic delays. Conversely, when basal ganglia or cortical signal abnormalities are detected in conjunction with parenchymal swelling, alternative diagnoses such as encephalitis or lymphoma should be considered, as parenchymal swelling is not a typical feature of sCJD as revealed by MRI.

## Introduction

Creutzfeldt-Jakob disease (CJD) is an uncommon and deadly spongiform encephalopathy triggered by the infectious prion protein. It is classified into four subtypes depending on its origin: familial, sporadic, variant, and iatrogenic.

Sporadic Creutzfeldt-Jakob disease (sCJD) develops when the normal prion protein PrPc transforms into the infectious PrPSc (scrapie isoform of the prion protein) form, and the precise mechanism of this transformation remains unknown [1]. It accounts for about 85 to 90 percent of all CJD cases and typically manifests around the age of 65 [2]. sCJD generally presents with rapid cognitive and functional decline, memory deficits, myoclonus, pyramidal and extrapyramidal symptoms, and visual disturbances. It is one of the important differential diagnoses for rapidly progressive dementia (RPD). These clinical features are used in the diagnostic criteria established by the World Health Organization (WHO), the MRI-CJD Consortium, and the 2018 CDC criteria for sCJD. Additional forms of CJD encompass familial CJD, characterized by a notable family history, variant CJD arising from the consumption of cattle infected with bovine spongiform encephalopathy, and iatrogenic CJD stemming from the use of contaminated medical equipment from a patient infected with CJD.

CJD is often missed as a diagnosis due to its infrequent and nonspecific symptoms. Furthermore, CJD may sometimes exhibit atypical clinical features that do not fit the established criteria, posing challenges for diagnosis. Hence, in addition to a comprehensive medical history and physical examination, it is crucial to use appropriate diagnostic tests such as blood and cerebrospinal fluid (CSF) analyses, neuroimaging, and electroencephalography (EEG) recording for periodic sharp wave complexes (PSWCs). These tests can help exclude other potential conditions while confirming the diagnosis of CJD.

Only a few case reports of Creutzfeldt-Jakob disease (CJD) have been previously documented in India. Notable cases include those reported by the National Creutzfeldt-Jakob Disease Registry at the National Institute of Mental Health and Neuroscience, Department of Neuropathology in Bangalore [3], and the Creutzfeldt-Jakob Disease Registry in Northern India, as outlined by Mehndiratta et al. [4]. This disease can be less reported and often goes unrecognized, primarily due to a lack of clinical awareness and the limited availability of genetic and microbiological testing.

The main purpose of this study is to investigate the incidence and diagnostic features of Creutzfeldt-Jakob disease, with a specific focus on cases encountered at our hospital over the past five years. The study offers further insights into this infrequent condition's clinical, imaging, and electroencephalographic characteristics.

This article was previously presented as a meeting poster at the 2021 AOCN Annual Scientific Meeting on April 1, 2021".

## Materials and methods

This study outlines the clinical, radiological, and electroencephalographic characteristics of Creutzfeldt-Jakob disease (CJD) cases observed at a tertiary care hospital in India between January 2017 and December 2021, spanning five years. The research, retrospective and observational, was conducted within a hospital setting. Following the Centers for Disease Control and Prevention's (CDC) latest diagnostic criteria from 2018, patients were categorized into sporadic, familial, variant, and iatrogenic types. Within sporadic CJD, further classifications included definite, probable, and possible subtypes.

The medical records of the included patients were thoroughly reviewed, and the following data points were collected: age, gender, occupation, diet, duration of symptoms when diagnosed, initial clinical presentation, subsequent symptoms or signs, history of any neurosurgical procedures or head trauma, past medical history, family history, and current medications. Complete blood counts including erythrocyte sedimentation rate, liver function tests, serum ammonia levels, renal function tests, serum electrolytes, serum calcium, vitamin B12 levels, thyroid function tests, antithyroid peroxidase antibody, ANA (antinuclear antibody status), human immunodeficiency virus (HIV 1 & 2) screening, serum VDRL (Venereal Disease Research Laboratory) titers, autoimmune encephalitis serum and CSF panel, including voltage-gated potassium channel (VGKC) antibody levels, and cerebrospinal fluid (CSF) analysis, including total cell count, differential cell counts, protein, glucose levels, and gram stain and AFB stain, bacterial culture and viral PCR panel for Herpes simplex virus were analyzed in the study.

The study employed a comprehensive approach to data collection and assessment for patients. Brain MRI was conducted using a 1.5 Tesla scanner, a standard strength for clinical imaging. Various MRI sequences were employed, including T1 and T2 weighted sequences, diffusion-weighted (DWI) sequences with ADC mapping, fluid-attenuated inversion recovery (FLAIR), and T1 post-contrast sequences. These sequences collectively provided a detailed evaluation of brain structure and potential pathology.

In addition to MRI, electroencephalogram (EEG) data was gathered using the international 10-20 system, a widely recognized electrode placement method in EEG recordings. To ensure the accuracy of interpretation, the EEG results were analyzed by a Neurologist with expertise in electrophysiology. The study specifically defined PSWCs in EEG recordings as complexes characterized by biphasic or triphasic sharp waves lasting for a minimum of 100 milliseconds and a maximum of 600 milliseconds, with recurrence at intervals of a minimum of 500 milliseconds. It can prolong up to a maximum of 2000 milliseconds. The investigative procedures did not include the CSF 14-3-3 protein assay or diagnostic brain biopsy. Instead, the study relied on non-invasive imaging and EEG assessments.

## Results

Over the five years, four cases were identified, and all were sporadic CJD cases. The patients exhibited a mean age of 67.5 years, falling within the age range of 57 to 75. Notably, all the patients were women. This study's observed incidence of CJD is 0.05 per one million people per year. However, caution is warranted in extrapolating this data, as no incidence data is available from India for comparison. All the cases were females. Familial CJD was deemed improbable in these cases, given the absence of a family history featuring similar symptoms. Variant CJD is also unlikely in these patients, as they exhibited a shorter duration of illness and lacked signature MRI findings. Iatrogenic CJD is ruled out, as these patients did not undergo any neurosurgical procedures. The primary clinical manifestations observed were dementia, which was rapidly progressive in nature, myoclonus, and extrapyramidal signs like rigidity, which were present in all four cases. Additionally, behavioral disturbances were noted in two cases, pyramidal signs in one case, ataxia in one case, akinetic mutism in one case, and visual disturbances in one case. The patients received a diagnosis of sporadic CJD, and their clinical and diagnostic features were summarized and presented in Tables 1-2.

**Table 1:** Demographic, Clinical, and Neuroimaging Profiling of Creutzfeldt-Jakob Disease Patients: A Comparative Analysis CDC, Centers for Disease Control and Prevention; No, number; F, female; EEG, electroencephalography; MRI, magnetic resonance imaging; PSWCs, periodic sharp wave complexes; N/A, not available

Case No	Age /Sex	Type	Symptoms at onset	Clinical features (symptoms and signs)	Duration of illness (months)	MRI	EEG	CDC Diagnostic Criteria	MRI Brain findings of each patient
1	71/F	Sporadic	Dementia	Myoclonus, Dystonia, Rapid Deterioration Of Consciousness	1 Month	Compatible	PSWCs	Probable	T2/FLAIR hyperintensity with diffusion restriction on DWI in the bilateral caudate, lentiform nucleus, bilateral parietal, right temporal, and right frontal cortex
2	58/F	Sporadic	Dementia	Myoclonus, Jaw and limb Dystonia, Rigidity, Brisk Reflexes, Cognitive Decline, Behavioral Disturbances, Akinetic Mutism, Vision Disturbances	3 Months	Compatible	Triphasic Waves	Probable	T2/FLAIR hyperintensity with diffusion restriction on DWI in bilateral caudate, lentiform nucleus, bilateral parietal lobes
3	75/F	Sporadic	Dementia	Myoclonus, Dystonia, Cognitive Decline	2 Months	Compatible	Diffuse Delta Slowing	Probable	T2/FLAIR hyperintensity with diffusion restriction on DWI in the bilateral caudate, lentiform nucleus, bilateral frontal, parietal, and left temporal cortex
4	67/F	Sporadic	Dementia	Ataxia, Rigidity, Behavioral Disturbances, Myoclonus, Rapid Deterioration of Consciousness	N/A	Compatible	Triphasic Waves	Probable	T2/FLAIR hyperintensity with diffusion restriction on DWI in the bilateral caudate, lentiform nucleus, thalamus, bilateral cingulate gyrus, and bilateral medial temporal lobes

**Table 2:** Comparing Clinical Characteristics of Creutzfeldt-Jakob Disease (CJD) Across Various Studies CDC, Centers for Disease Control and Prevention

Clinical characteristics	Present series (n=4)	Mehindiratta et al., 2001 [4] (n=10)	Velasquez-Perez et al., 2007 [5] (n=15)	GonzMez-Duarte et al., 2011 [6] (n=7)
Mean age in years (range)	67.5 years (57-75 years)	53.8 (42-60)	49 (23-76)	55 (38-66)
Gender (female) (n)	4	5	3	0
CDC Diagnosis				
Definite	0	2	3	0
Probable	4	7	5	5
Possible	0	1	7	2
Clinical symptoms				
Dementia	4	6	12	4
Behavioral disturbances	2	7	6	3
Psychosis	0	1	4	2
Ataxia	1	2	5	7
Myoclonus	4	10	6	3
Extrapyramidal symptoms	4	5	9	3
Pyramidal symptoms	1	2	4	2
Hallucinations	0	5	3	3
Akinetic mutism	1	1	7	5
Aphasia	0	0	1	0
Cortical blindness	1	1	-	-
Tremor	1	1	1	3
Sleep disturbances	2	4	1	3
Interval between onset-admission	2.7 months	4.5 months (1-18)	71 days (10-210)	64 months (2-12)
Interval between onset-death	6.6 months	66 months (1-18	-	-
Family history	0	1	0	2

At symptom onset, all patients exhibited progressive cognitive decline. Myoclonus and extrapyramidal symptoms (rigidity) were present in all cases upon admission. The mean duration between illness onset and admission to the hospital was 2.7 months, ranging from 1 to 5 months. At the time of admission, all patients were bedridden. Between the onset of symptoms and death, the mean interval duration was 6.6 months, ranging from 3 to 8 months.

**Figure 1:**
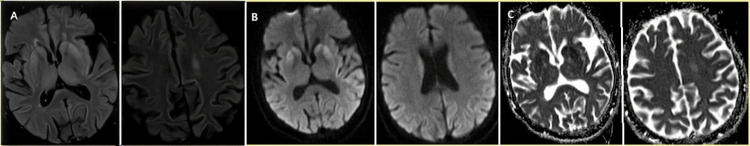
Magnetic resonance brain images of a patient with Creutzfeldt-Jakob disease: Changes in deep gray matter and bilateral temporo-parietal regions MRI BRAIN FLAIR, DWI, and ADC axial sequences of patient 3 are shown. FLAIR sequence (first 2 images on the right (A)) shows hyperintensities in the bilateral caudate, putamen, and thalamus with diffusion restriction on DWI&ADC mapping (corresponding DWI sequences in the 2 images on the middle (B), corresponding ADC in the two images on the left (C)). FLAIR reveals hyperintensities in bilateral caudate and putamen regions with diffusion restriction apparent on DWI and ADC mapping. Similar FLAIR hyperintensities with diffusion restriction are also noted in bilateral parasagittal frontal and parietal lobes, primarily on the left side, and in the left insular cortex. Notably, the cortical diffusion restriction exhibits a cortical ribboning pattern. MRI, magnetic resonance imaging; FLAIR, fluid-attenuated inversion recovery; DWI, diffusion-weighted imaging; ADC, apparent diffusion coefficient.

**Figure 2:**
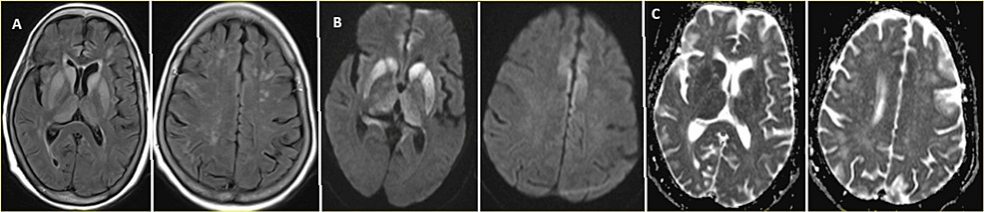
Magnetic resonance brain images of a patient with Creutzfeldt-Jakob disease: changes in deep gray matter and bilateral parasagittal regions MRI BRAIN FLAIR, DWI, and ADC axial sequences of patient 4 are shown. FLAIR sequence (first two images on the right (A)) shows hyperintensities in the bilateral caudate, putamen, and thalamus with diffusion restriction on DWI&ADC mapping (corresponding DWI sequences in the two images in the middle (B), corresponding ADC in the two images on the left (C)). Similar FLAIR hyperintensities with diffusion restriction were also noticed in bilateral cingulate gyrus Cortical diffusion restriction has cortical ribboning pattern. FLAIR hyperintensities with no diffusion restriction notes in subcortical and deep white matter of frontal and parietal lobes suggestive of probable small vessel/microvascular ischemic changes. MRI, magnetic resonance imaging; FLAIR, fluid-attenuated inversion recovery; DWI, diffusion-weighted imaging; ADC, apparent diffusion coefficient.

Brain MRI abnormalities were observed in all patients, characterized by T2 and FLAIR hyperintensities and diffusion restriction in the corresponding regions on diffusion-weighted images with apparent diffusion coefficient mapping (DWI/ADC) in the caudate, putamen, and thalamus. Furthermore, a cortical ribbon sign with diffusion restriction was noted on the frontal, parietal, and temporal lobes. A summary of the detailed brain MRI characteristics for each patient is presented in Table 2, and the brain imaging abnormalities of two patients are shown in Figures 1-2. 

The main electroencephalography (EEG) findings included generalized delta slow waves with recurrent triphasic complexes in three of the four cases. One case exhibited classical PSWCs occurring at a frequency of one per 1000 milliseconds. The electroencephalography (EEG) findings of two patients diagnosed with Creutzfeldt-Jakob Disease (CJD) are as follows. Patient 1 exhibited background activity of 15-16 Hz with occasional delta slow waves. Notably, there were periodic intervals of generalized frontally dominant slow waves with a triphasic morphology.

On the other hand, Patient 2 displayed background activity featuring theta (4-7 Hz) and beta (15-16 Hz) waves, along with recurrent generalized slow waves characterized by a triphasic morphology. These EEG findings are indicative of the characteristic abnormalities seen in CJD patients, emphasizing the importance of EEG in diagnosing this rapidly progressive neurodegenerative disorder. Patient 3's EEG displayed a background activity characterized by 15-16 Hz beta waves and low-amplitude beta activity ranging from 10-20 microvolts. Additionally, intermittent episodes of generalized slow waves with a frontal emphasis were observed. In the case of patient four, the EEG exhibited background activity with a combination of theta waves, ranging from 4-7 Hz and measuring 40-70 microvolts, along with beta activity at 15-16 Hz with amplitudes of 10-20 microvolts. Periodic instances of generalized slow waves with frontal predominance were noted intermittently with triphasic morphology. In all cases, cerebrospinal fluid (CSF) analysis revealed normal protein levels, cell counts within normal limits, and negative bacteriological and viral findings. The CDC diagnostic criteria have been applied to these four cases, and the results (Table 3) indicate probable sporadic Creutzfeldt-Jakob disease.

**Table 3:** Comparing EEG and radiological features of Creutzfeldt-Jakob Disease (CJD) across various studies EEG, Electroencephalography; CT, Computed Tomography; MRI, Magnetic Resonance Imaging, PLEDS, periodic lateralized epileptiform discharges

	Present series (n=4)	Mehindiratta et al., 2001 [4] (n=10)	Velasquez-Perez et al., 2007 [5] (n=15)	GonzMez-Duarte et al., 2011 [6] (n=7)
EEG Findings				
PSWC	1	6	12	4
TW	4	3	2	2
Diffuse slowings	4	0	0	1
PLEDS	0	0	0	0
N/A	0	1	0	0
CT/MRI Findings				
	MRI	CT	MRI	MRI
Basal Ganglia and Cortical Ribboning	4	0	6	6
Atrophy	0	3	2	1
Normal	0	7	2	0
White matter lesions	1	0	1	0
N/A	0	0	4	0

## Discussion

Study participants had a mean age of 67.5 years, similar to the mean age reported in the existing literature for sporadic CJD [4,5]. A noteworthy observation was the female predominance among the patients, consistent with findings from other published case series [4-6]. The observed incidence of CJD in this study, at 0.05 per one million population per year, is notably lower than the incidence rates of 0.5 to 1.5 cases per million per year reported in the existing literature [4]. However, caution is warranted in extrapolating this data, as no incidence data is available from India for comparison. It is essential to emphasize that in India, CJD remains significantly underreported. As of September 2005, the national CJD registry had recorded merely 85 documented cases of CJD [3]. It is noteworthy that demographic characteristics in these cases were found to be in line with reports from other regions worldwide. A north India study reported an incidence of 10 cases over nine years (1990-1998) [4]. A similar study from eastern India reported an incidence of 10 cases over three years (2011-2013) [7]. Given these factors and the rarity of CJD, it is remarkable that our center, which serves a population of approximately 15 million individuals in the Rayalaseema region of South India, was able to identify and register four cases within a five-year timeframe. This highlights the need for continued research and awareness regarding CJD, especially in regions where it may be underreported. The last sporadic CJD study done in India was in 2013, with gaps in surveillance programs.

All patients presented with rapidly progressive dementia accompanied by myoclonus. Additional clinical features included behavioral disturbances, ataxia, and extrapyramidal symptoms. Several clinical observations agree with previous case reports, suggesting that the presentation of sCJD is consistent with previous reports [5,6]. All the patients were investigated for differential diagnoses like infections, autoimmune diseases, paraneoplastic diseases, and brain mass lesions using relevant investigations.

The presence of behavioral disturbances, a hallmark of CJD, were found in 50% of patients at the time of onset, which is within the range reported in the literature. The absence of a family history of CJD strongly suggests that the disease is sporadic rather than familial [8]. There was a significant difference between the time interval between symptom onset and diagnosis, with a median duration of 2.7 months, ranging from 30 days to 5 months, consistent with findings in other case series. The mean duration from symptom onset to death, averaging 6.6 months, corresponds with survival patterns commonly observed in sporadic CJD patients [3-6,8].

Neuroimaging plays a pivotal role in diagnosing and understanding Creutzfeldt-Jakob disease (CJD). Key findings include high signal intensities in T2-weighted and FLAIR sequences, indicating astrocytic gliosis, and distinct patterns of abnormality in DWI and FLAIR sequences, notably combined cortical and deep gray matter hyperintensity or isolated cortical involvement [9]. Neuronal loss, gliosis, and spongiform change are the salient neuropathogenic changes in sCJD. Cytotoxic oedema secondary spongiform change constituted by intracytoplasmatic vacuoles has been noted in T2/DW1 hyperintensity areas [9]. DWI emerged as a sensitive tool, surpassing FLAIR in the early detection of cortical abnormalities.

It is important to note that DWI has a greater specificity (93%) than sensitivity (92%) for diagnosing CJD, regardless of the signature EEG changes [10]. Additionally, deep gray matter involvement, like the basal ganglia, is associated with an accelerated disease course [11]. In prior studies, including the study conducted by Biswas et al. in 2013, abnormalities have been reported in all CJD patients, with bilateral FLAIR hyperintensities in the basal ganglia and diffusion restriction in various cortical regions being specific findings [12]. In neuroimaging, combining DWI with FLAIR sequences has been shown to increase the specificity (95%) for diagnosing CJD [7]. By combining these two approaches, we can differentiate sporadic CJD from other RPDs with improved sensitivity and specificity, as reported by Vitali et al. in 2011 [13]. This comprehensive neuroimaging approach aids CJD diagnosis, guides patient management, and distinguishes CJD from other RPDs, enhancing our understanding of this devastating neurological disorder, particularly in resource-poor settings.

PSWCs and triphasic waves, the classical EEG changes, were consistently observed in all the study participants. EEG demonstrates a moderate sensitivity of 67% and a relatively higher specificity ranging from 74% to 86% in CJD diagnosis [5]. This suggests that while EEG is valuable for detecting CJD, false-negative results may occur, necessitating a comprehensive diagnostic approach. The study highlights the need for repeated EEGs during the progression of the disease, especially if the initial recordings are not specific, as the changes evolve over time. Multiple EEG assessments increase the likelihood of capturing characteristic EEG abnormalities associated with CJD [14]. This emphasizes the dynamic nature of EEG findings in the context of disease progression. Notably, EEG abnormalities such as PSWC and triphasic waves are rarely observed in common forms of dementia, including vascular dementia and Alzheimer's disease, and reversible dementias like autoimmune encephalitis.

All the study participants hailed from the Rayalaseema area of Andhra Pradesh, India, suggesting a notable prevalence of CJD in this specific region. The study highlights the importance of referring patients to specialized neuroscience centers. It underscores the need to enhance CJD surveillance in India, particularly by improving access to advanced diagnostic tests.

The study was limited by the absence of postmortem brain examinations to confirm the diagnosis of CJD. The gold standard for definitive diagnosis is histological examination and immunostaining for protease-resistant protein (PrPSc) in brain tissue. It should be noted, however, that without this confirmation, the cases remain suggestive of probable prionopathy and lack conclusive histological evidence. The study could not conduct PrP genotyping or analyze the patients' prion protein gene (PRNP) polymorphism. The genetic information is crucial since differences in codon 129 polymorphisms and PrPSc typing contribute to the clinical heterogeneity of sporadic CJD. It may be difficult to characterize these patients' diseases comprehensively because genetic data is lacking. A further limitation of this study was the inability to perform the 14-3-3 protein assay in these patients. CJD diagnosis may have been impacted by the absence of this assay in some cases. Reasons cited include financial constraints, a lack of local expertise, and logistical challenges related to transferring samples to foreign laboratories.

## Conclusions

Sporadic CJD remains a challenging diagnosis due to its rarity and atypical clinical features. This study reinforces the importance of a comprehensive diagnostic approach for accurate diagnosis, including neuroimaging and EEG. The observed patterns in clinical presentation, imaging, and EEG findings align with existing literature and contribute valuable data to understanding sporadic CJD. Additionally, improving access to advanced diagnostic tools and increasing CJD surveillance are crucial steps in enhancing our understanding of this devastating neurological disorder, especially in regions where awareness and resources are limited. The limitations related to the lack of postmortem brain examination and 14-3-3 protein assay should be considered when interpreting the findings. With the availability of these diagnostic tools, future studies may better understand sporadic CJD and its clinical heterogeneity.
